# Modified Proximal Gastrectomy and D2 Lymphadenectomy Is an Oncologically Sound Operation for Locally Advanced Proximal and GEJ Adenocarcinoma

**DOI:** 10.3390/cancers17152455

**Published:** 2025-07-24

**Authors:** Emily L. Siegler, Travis E. Grotz

**Affiliations:** Department of Surgery, Mayo Clinic, Rochester, MN 55905, USA; siegler.emily@mayo.edu

**Keywords:** gastric cancer, gastroesophageal cancer, proximal gastrectomy, double tract reconstruction, D2 lymphadenectomy

## Abstract

**Background:** Proximal gastrectomy (PG) with double tract reconstruction (DTR) offers organ preservation for early gastric cancers, leading to reduced vitamin B12 deficiency, less weight loss, and improved quality of life. The JCOG1401 study confirmed excellent long-term outcomes for PG in stage I gastric cancer. However, in locally advanced proximal gastric cancer (LAPGC), preserving the gastric body and lymph node station 4d may compromise margin clearance and adequate lymphadenectomy. **Methods**: We propose a modified PG that removes the distal esophagus, gastroesophageal junction (GEJ), cardia, fundus, and gastric body, preserving only the antrum and performing DTR. Lymphadenectomy is also adapted, removing stations 1, 2, 3a, 4sa, 4sb, 4d, 7, 8, 9, 10 (spleen preserving), 11, and lower mediastinal nodes (stations 19, 20, and 110), while preserving stations 3b, 5, and 6. Indications for this procedure include GEJ (Siewert type II and III) and proximal gastric cancers with ≤2 cm distal esophageal involvement and ≤5 cm gastric involvement. **Results:** In our initial experience with 14 patients, we achieved R0 resection in all patients, adequate lymph node harvest (median 24 nodes, IQR 18–38), and no locoregional recurrences at a median follow-up of 18 months. We also found favorable postoperative weight loss, reflux, and anemia in the PG cohort. **Conclusion:** While larger studies and long-term data are still needed, our early results suggest that modified PG—despite sparing only the antrum—retains the key benefits of PG over total gastrectomy, including better weight maintenance and improved hemoglobin levels, while maintaining oncologic outcomes for LAPGC.

## Simple Summary

Gastric cancer patterns have changed in the US and Western Europe, with more cases now occurring in the upper stomach. While surgery remains the main treatment, removing the entire stomach (total gastrectomy) can lead to long-term issues like weight loss and nutritional problems. This study looked at an alternative, more stomach-sparing surgery called *proximal gastrectomy (PG)* with *double tract reconstruction (DTR)* for patients with upper stomach cancers. Fourteen patients at the Mayo Clinic had this surgery and were compared to others who had more extensive stomach removals. PG patients had similar survival rates, recovery, and complication rates, but showed signs of better weight maintenance and higher blood levels (hemoglobin), possibly due to preserved stomach function and improved nutrient absorption. The findings suggest PG with DTR may be a safe and effective option for certain patients with tumors limited to the upper stomach and with specific criteria (like tumor size and location). This approach may improve quality of life after surgery, but larger studies are needed to confirm these early results and refine patient selection.

## 1. Introduction

Over the past several decades, there has been an epidemiologic shift in gastric cancer in the United States and Western Europe with an increase in the incidence of diffuse-type histology and proximal stomach location, particularly at the gastroesophageal junction and cardia. Simultaneously, there has been a marked decline in intestinal-type gastric cancer and distal stomach location. This trend contrasts with global patterns where distal gastric tumors remain more common, particularly in regions with high H. pylori prevalence. The increased prevalence of diffuse-type gastric cancer is attributable to the increased incidence of obesity, hypergastrinemia, and gastroesophageal reflux in Western countries, as well as the simultaneous success of public health measures resulting in increased access to clean drinking water, improved food preservation, and eradication of H. pylori. Despite these changes in tumor location, gastric cancer in the United States and Western Europe is still frequently diagnosed at an advanced stage, reflecting the lack of widespread screening programs and the often asymptomatic nature of early-stage gastric cancer, leading to delays in diagnosis [1]. Locally advanced proximal gastric cancer (LAPGC) presents a unique challenge due to the anatomical location and complexity of surgical resection. Although surgery remains the cornerstone of curative therapy for gastric cancer, perioperative therapies such as chemotherapy and radiation can improve survival outcomes in LAPGC. Herein, we examine the data on proximal gastrectomy (PG) and lymphadenectomy to propose evidence-based criteria for patient selection, standardized proximal and distal transection points, and clear definitions of lymphadenectomy while also sharing our initial experience with PG for LAPGC.

## 2. Methods

This study protocol was reviewed and approved by the Institutional Review Board (IRB) (24-009946) at our institution and was conducted in accordance with the principles of the Declaration of Helsinki and its later revisions. All patients signed the Minnesota Research Authorization (MRA) and signed informed consent for the operation. We subsequently and retrospectively included 14 patients with locally advanced (≥T2 or N+) proximal gastric cancer at the Mayo Clinic Rochester between December 2022 and October 2024. All patients underwent EUS staging and were found to have ≤2 cm of distal esophageal involvement and ≤5 cm of proximal gastric involvement. They also did not have any evidence of station 5 or 6 lymphadenopathy on cross-sectional imaging and/or EUS. All patients underwent a staging laparoscopy with peritoneal cytology and received perioperative systemic chemotherapy and underwent robotic PG with double tract reconstruction (DTR). A control group of 88 patients with locally advanced gastric cancer who underwent minimally invasive (MIS) total gastrectomy (TG) or subtotal gastrectomy (SG) at our institution between April 2003 and June 2023 was also included for comparison.

### 2.1. Surgical Technique

**Figure 1:**
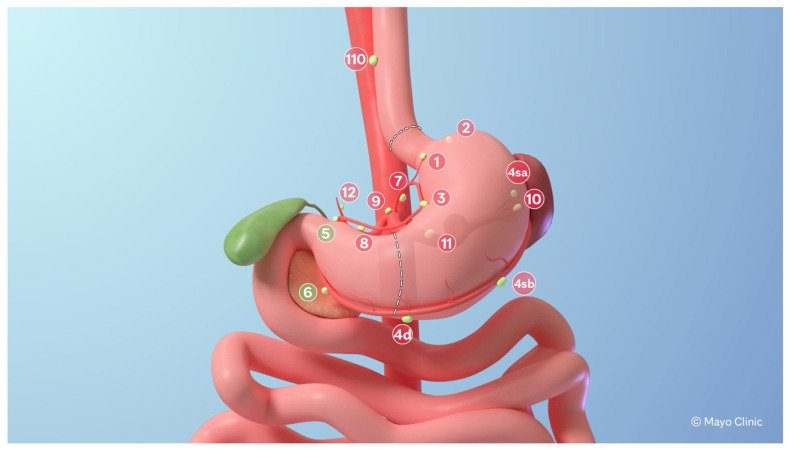
Schematic of proposed proximal gastrectomy with distal transection point at the incisura angularis and the proximal transection point on the distal esophagus with modified D2 lymphadenectomy to include stations (marked in red) 1, 2, 3, 4sa, 4sb, 4d, 7, 8, 9, 10, 11, 12, and 110. Stations 5 and 6 (marked in green) are spared along with the antrum.

**Figure 2:**
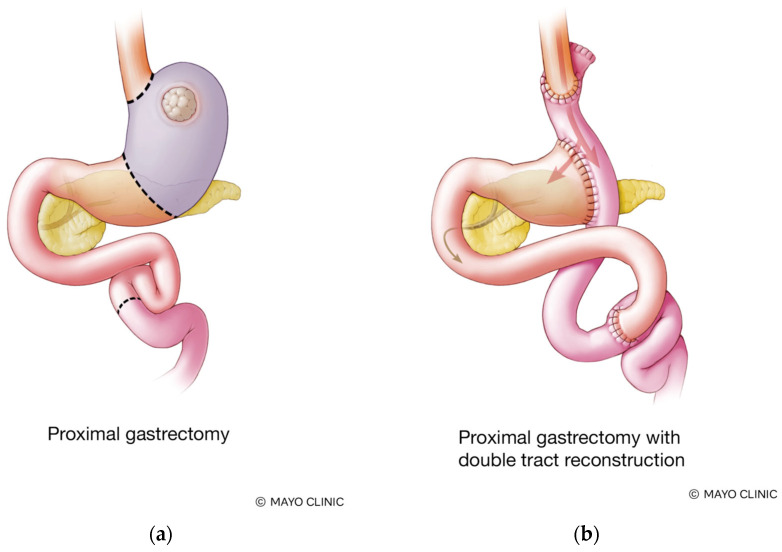
(**a**) Normal foregut anatomy, (**b**) after proximal gastrectomy (with resection of the distal esophagus, cardia, fundus, and body) and double tract reconstruction.

All patients underwent robotic PG (Da Vinci Xi, Intuitive) with transection of the stomach vertically at the incisura angularis and had a modified D2 lymphadenectomy with stations 1, 2, 3a, 4s, 4p, 4d, 7, 8, 9, 10 (spleen preserving), 11, 19, 20, and 110 removed (Figure 1). The spleen was preserved during station 10 nodal dissection in all patients. The omentum was divided in half, and the left-sided omentum adjacent to the proximal stomach was resected en bloc. DTR was performed with a retrocolic Roux limb and an intracorporal hand-sewn esophagojejunostomy (E–J) with interrupted sutures. Approximately 10–15 cm distal to the E-J anastomosis, we created an end-to-side, hand-sewn gastrojejunostomy (G–J) with the antrum and the Roux limb using a running barbed suture. We then measured a 20–25 cm infracolic Roux limb and created a side-to-side stapled jejuno-jejunostomy anastomosis (Figure 2). All mesenteric defects were closed with a permanent suture. The antrum was tacked to the right diaphragmatic crus to maintain a horizontal orientation.

### 2.2. Data Analysis

Descriptive statistics were reported as median and interquartile range (IQR) or number and frequency. Chi-square test was used to compare categorical variables between groups, and one-way ANOVA was used to compare continuous variables among groups. The mean percentage of body weight loss was recorded at 6 and 12 months +/− 4 weeks after surgery. We assumed the missing data were missing at random and thus performed pairwise deletion of missing data.

## 3. Results

### 3.1. Baseline Characteristics

During the study period, 14 patients underwent robotic PG with DTR, 54 underwent laparoscopic or robotic TG, and 34 underwent laparoscopic or robotic SG. There were no significant differences in age, sex, BMI, or clinical stage among the groups (Table 1). Differences in histologic subtype and tumor grade were observed, though these were partially influenced by missing data. Variations in perioperative treatment (chemotherapy, radiation, and chemoradiation) were also noted and appeared to reflect differences in tumor location, with gastroesophageal junction (GEJ) tumors more commonly seen in the PG group, leading to more frequent use of chemoradiation within this cohort.

### 3.2. Perioperative Outcomes

Postoperative outcomes were comparable across all three cohorts (Table 2). An R0 resection was achieved in all PG cases. The median number of lymph nodes removed was not significantly different among the groups: 24 (IQR 18–38) for PG, 29 (IQR 20–38) for TG, and 30 (IQR 21–40) for SG (*p* = 0.90). Operative time, estimated blood loss, and length of stay were also similar between cohorts. No PG patients required postoperative transfusion. While none of the PG patients received enteral nutrition postoperatively, two patients (14%) required temporary parenteral nutrition within 30 days after surgery (Clavien–Dindo grade II). Thirty-day readmission rates did not differ significantly: 14% for PG, 13% for TG, and 24% for SG (*p* = 0.4). The two readmissions of patients who underwent PG were for dysphagia necessitating parenteral nutrition (Clavien–Dindo grade II), and for gastrointestinal bleeding of indeterminate source that was managed conservatively without necessitating transfusion or endoscopic evaluation (Clavien–Dindo grade I). Major morbidity within 90 days was also comparable: 14% for PG, 26% for TG, and 12% for SG (*p* = 0.28). No anastomotic leaks occurred. The two major complications in the PG cohort were GJ ulcer discovered on esophagogastroduodenoscopy (EGD) performed for persistent vomiting, and benign stricture requiring dilation (both Clavien–Dindo IIIa complications). Ninety-day mortality was 0% for PG and TG and 3% (1 patient) for SG (*p* = 0.34). No PG patients required reoperation within 30 days, compared to eight patients (15%) in the TG group and three patients (9%) in the SG group (*p* = 0.24).

### 3.3. Biliary Reflux

**Figure 3:**
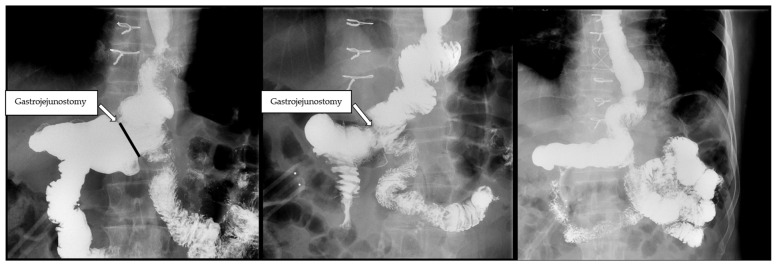
Postoperative fluoroscopy demonstrating equal passage of contrast through the gastroduodenal limb and Roux limb.

In postoperative clinical follow-up, one patient developed bile reflux symptoms significant enough to warrant endoscopy that demonstrated LA Grade B esophagitis (Clavien–Dindo grade IIIa). Three patients reported mild biliary reflux symptoms that were well-controlled with anti-reflux medications (Clavien–Dindo grade I). Upper gastrointestinal contrast studies, performed only in patients who were symptomatic with either reflux or dysphagia postoperatively, demonstrate passage of contrast equally through the gastroduodenal limb as well as the Roux limb (Figure 3). There were no reports of delayed gastric emptying in our cohort, nor was this demonstrated in any postoperative workup conducted for reflux.

### 3.4. Oncologic Outcomes

At a median follow-up of 18 (IQR 14–23) months, no locoregional recurrences were observed in the PG group. The 18-month overall survival rate was 86%; of the two deaths, one was due to distant metastasis and the other resulted from complications of an unrelated traumatic fall.

### 3.5. Weight Loss

**Figure 4:**
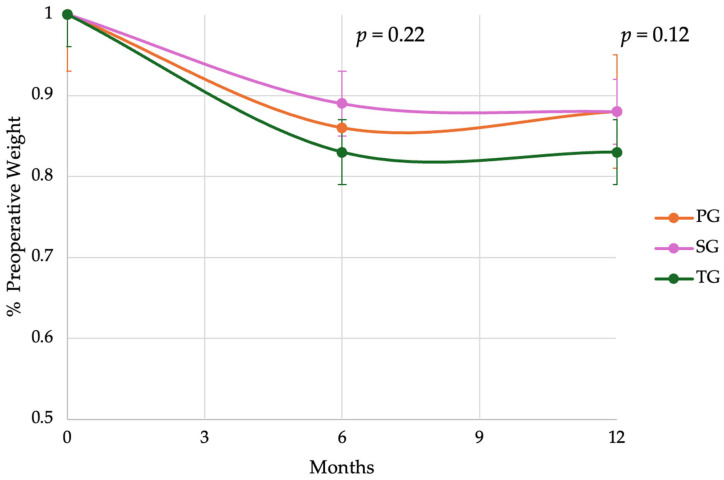
Postoperative weight loss is reported as the mean percentage of actual body weight loss over the first 12 months after surgery for patients undergoing proximal gastrectomy (PG), total gastrectomy (TG), and subtotal gastrectomy (SG). Statistical significance and confidence intervals are included, calculated for 95% confidence from the mean percentage preoperative weight at 12 months.

In our preliminary PG cohort, the mean weight loss at 6 months was 14%, which was comparable to the 12% in the SG and 17% in the TG cohort (*p* = 0.22). By 12 months, patients in the PG cohort had regained some weight, resulting in a mean weight loss of 12%, aligning more closely with the SG cohort (12%) and diverging from the continued weight loss observed in the TG cohort (17%), although this difference was not of statistical significance (*p* = 0.12) (Figure 4). We suspect these findings are underpowered due to the small sample size and anticipate that with increased experience and a larger PG cohort, the difference in weight trajectories between PG and TG will become more evident. Physiologically, the weight stabilization observed in PG is consistent with the preservation of the antrum as a gastric reservoir and the absorptive advantage provided by the DTR. These observations are also consistent with clinical experience, particularly in the early postoperative period, where the E–J (in both the TG and PG) may limit caloric intake and can be plagued by strictures.

### 3.6. Anemia and Postoperative Hemoglobin

**Figure 5:**
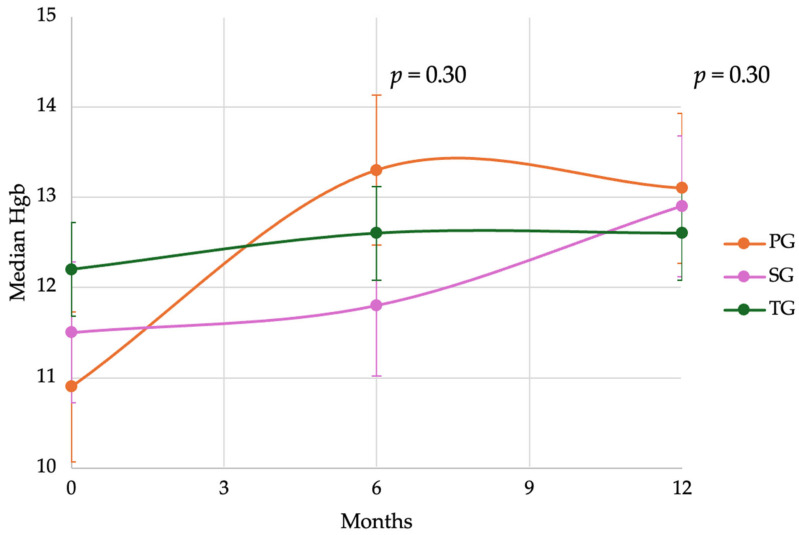
Postoperative iron deficiency anemia was reported as median hemoglobin (Hgb) at 6 and 12 months for patients undergoing proximal gastrectomy (PG), total gastrectomy (TG), and subtotal gastrectomy (SG). Statistical significance and confidence intervals are included, calculated for 95% confidence from median Hgb at 12 months.

We evaluated postoperative hemoglobin (Hgb) levels at 6 and 12 months in patients undergoing PG, TG, and SG. Despite a lower baseline Hgb level in the PG cohort compared to the TG and SG cohorts, the PG group consistently demonstrated higher median values postoperatively (Figure 5). At 6 months, the median Hgb levels were 13.3 g/dL (IQR 12.6–13.5) for PG, 12.6 g/dL (IQR 10.8–13.2) for TG, and 11.5 g/dL (IQR 10.9–12.8) for SG. Using anemia thresholds of <13 for men and <12 for women, anemia rates were 45.4% (5/11 patients) for PG, 62.9% (22/35) for TG, and 55.6% (10/18) for SG. At 12 months, the median Hgb levels were 13.1 g/dL (IQR 11.6–14.8) for PG, 12.6 g/dL (IQR 11.7–13.4) for TG, and 12.9 g/dL (12.3–13.8) for SG. While these findings did not reach statistical significance, the trend toward higher median hemoglobin levels in the PG group suggests a potential benefit of improved iron absorption in the DTR. However, the clinical significance of this difference in Hgb remains undefined. We suspect the current analysis is underpowered, and that larger studies may reveal a more definitive benefit associated with PG in preserving the postoperative nutritional and hematologic status.

## 4. Discussion

### 4.1. Oncologic Safety of Partial Gastrectomy

The primary aim of surgical resection in gastric cancer is to achieve a complete tumor clearance with negative margins (R0). Due to the infiltrative nature of the malignancy, a grossly negative macroscopic margin is essential to account for potential microscopic submucosal spread. Once the clear margin is achieved, more extensive resections do not offer additional oncologic benefit. This was demonstrated in a randomized trial comparing TG and SG for distal gastric cancers, which showed no improvement in outcomes with TG when a macroscopic proximal margin ≥ 6 cm was achieved [2]. Although this study predated perioperative therapy, more recent data indicate that within the context of perioperative chemotherapy, a macroscopic proximal margin of ≥3 cm, combined with intraoperative frozen section confirmation, provides high oncologic safety and minimizes the risk of R1 resections [3]. Similarly, a US multi-institutional study found no correlation between wider microscopic margins and improved local control or overall survival [4]. This principle also applies to proximal gastric cancer. A large retrospective study from Memorial Sloan Kettering Cancer Center dating back to 1998 showed that proximal gastrectomy (PG) offers oncologically equivalent outcomes to TG in appropriately selected patients [5]. However, PG with esophago-gastrostomy resulted in unsatisfactory outcomes in terms of bile and acid reflux, with up to 87% of patients experiencing bile or acid reflux and was thus largely abandoned in the US [6]. As a result, TG remains the standard surgical treatment for LAPGC by default in the US and Western Europe.

The recent KLASS 5 randomized trial found no significant difference in short-term outcomes—including operative time, estimated blood loss, morbidity, mortality, or two-year disease-free and overall survival between laparoscopic PG with DTR compared to laparoscopic TG for early proximal gastric cancers [7,8]. A subsequent meta-analysis by Zhu et al. confirmed comparable five-year survival rates between the two operations, firmly supporting PG as an oncologically sound operation for early-stage proximal gastric cancer [9]. However, the evidence is less conclusive for LAPGC. Two independent propensity score-matched analyses, one from China and the other from Korea, found no significant difference in 3-, 5-, and 10-year recurrence-free or overall survival between PG and TF in patients with LAPGC [9,10]. Nonetheless, data from Western populations remain limited, and there is still no standardized approach in the US to PG for LAPGC.

### 4.2. Benefits of Preserving Distal Stomach in PG

TG has significant postoperative drawbacks. Patients undergoing TG frequently experience substantial postoperative weight loss, with a large study from Memorial Sloan Kettering Cancer Center indicating a mean weight loss rate of around 20% that persists for at least two years post-surgery [11]. Additionally, TG is associated with a higher risk of nutritional deficiencies—most notably, vitamin B12 deficiency—due to the loss of intrinsic factor production by the stomach [12,13]. In 1988, Aikou et al. introduced PG with antral preservation and double tract reconstruction (DTR) as an alternative surgical approach [14]. DTR involves three anastomoses: an esophagojejunostomy, a gastrojejunostomy, and a jejuno-jejunostomy. Importantly, this reconstructive method resulted in significantly lower rates of reflux esophagitis and anastomotic stricture compared to esophagogastrostomy reconstruction, with only 7.6% of patients experiencing reflux [14]. A subsequent prospective trial by Li et al. randomized 300 cTl-3N0M0 Siewert type II and III gastroesophageal cancers to PG or TG. PG resulted in significantly less reflux esophagitis than TG with a Roux-en-Y reconstruction [15]. This surgical technique has been further refined in Korea and Japan to preserve the body of the stomach, thereby maintaining intrinsic factor production, resulting in improved Hgb levels and reduced need for B12 replacement therapy in randomized clinical trials compared to total TG [9]. The preservation of the distal stomach in PG has also been associated with significantly less weight loss (9.6% versus 17.9%) and reduced skeletal muscle loss (9.3% vs. 18.3%) compared to TG [16]. The above randomized trial by Li et al. reported improved postoperative outcomes following PG, including higher albumin levels, improved weight maintenance, and higher vitamin B12 levels compared to TG [15].

Moreover, a prospective multi-institutional study found that patients undergoing PG for early gastric cancer experienced better quality of life with improved scores for indigestion and overall symptoms, compared to those who underwent TG [17]. These findings support preservation of the distal stomach as a key surgical objective given its association with improved postoperative nutritional parameters and more favorable digestive function, attributable to the maintenance of the gastric reservoir capacity and more physiologic duodenal passage of enteral nutrition. Currently, the Japanese Gastric Cancer Treatment Guidelines (JGCTG) (6th edition) recommend PG only for cT1N0 gastric cancers, where at least half of the distal stomach (body and antrum) can be preserved [18]. For more advanced proximal gastric cancers, a more radical PG and lymphadenectomy may be necessary.

### 4.3. Proposed Standardization of PG with DTR and D2 Lymphadenectomy for LAPGC

While PG is the standard treatment for cT1N0 proximal gastric cancer in Japan, its application to LAPGC in Western countries raises concern, primarily regarding the ability to achieve an adequate negative margin while preserving the distal stomach. In the US, the mean primary gastric tumor size is 4–5 cm, and a gross margin of at least 3 cm, confirmed by intraoperative frozen section, is recommended to ensure oncologic adequacy and reduce local recurrences [3,19]. Thus, securing a sufficient negative margin is essential when considering organ-preserving surgery for LAPGC. Anthropomorphic studies reveal that the mean lengths of the lesser and greater curvatures of the stomach are approximately 16 cm and 22 cm, respectively. Including the body of the stomach in the PG allows for a better distal margin as the gastric antrum typically spans approximately 4–6 cm along the lesser curvature and 5–8 cm along the greater curvature. Vertical transection at the angular incisure removes the body, fundus, and cardia of the stomach. This results in approximately 10 cm of proximal stomach along the lesser curvature and 15 cm along the greater curvature—sufficient to ensure an adequate distal margin for LAPGC extending ≤ 5 cm into the proximal stomach. This tumor size threshold is clinically relevant, as tumors larger than 5 cm are associated with increased risk of metastasis to lymph node stations 5 and 6 [20]. Accordingly, PG with DTR is best reserved for LAPGC patients with tumors involving ≤ 5 cm of the proximal stomach.

Regarding the proximal margin, current evidence supports the safe resection of proximal gastric tumors with ≤2 cm of esophageal involvement using a transhiatal approach. Hurata et al. demonstrated that GEJ cancers extending up to 2 cm into the esophagus can be resected with negative margins via a robotic transhiatal technique [21]. Similarly, Brown et al. concluded that a transabdominal extended TG could achieve a satisfactory proximal margin in selected patients with ≤2 cm of esophageal extension [22]. Importantly, esophageal involvement beyond 2 cm is associated with increased risk of mediastinal lymph node metastasis, making ≤ 2 cm of esophageal involvement a key criterion for selecting patients with LAPGC for PG [20]. In clinical practice, the Siewert classification—based on tumor epicenter—is less useful in guiding the operative approach than measuring the distance of tumor extension above and below the GEJ. For PG in LAPGC, the proximal transection should be 2–4 cm above the GEJ, tailored to the extent of esophageal involvement.

D2 lymphadenectomy has become the standard of care for oncologic lymphadenectomy in Western countries following the results of the Dutch D1D2 trial. This study demonstrated significantly lower rates of local (12% vs. 22%) and regional (13% vs. 19%) recurrence in the D2 dissection compared to those who had a D1 dissection [23]. These benefits translated to improved cancer-specific survival for the D2 group. However, overall survival was not improved, largely due to the increased perioperative mortality associated with routine distal pancreatectomy and splenectomy performed to clear lymph node stations 10 and 11 [23]. A subsequent multicenter prospective trial in Italy reinforced these findings, demonstrating that D2 lymphadenectomy provides excellent long-term survival and acceptable postoperative morbidity and mortality, particularly when a distal pancreatectomy and splenectomy are avoided [24]. The definition of a D2 lymphadenectomy depends on the type of gastrectomy performed. According to the JGCTG (6th edition), for TG, a D2 lymphadenectomy includes stations 1, 2, 3, 4, 5, 6, 7, 8a, 9, 11p, 11d, and 12a [18]. In contrast, for SG, stations 2 and 11d are omitted to preserve the posterior short gastric vessels, which are essential for maintaining adequate blood supply to the gastric remnant [18]. A theoretical concern with PG is the potential for inadequate lymphadenectomy, as it does not encompass the same extent of nodal dissection as TG. According to the JGCTG (6th edition), D2 lymphadenectomy for PG includes stations 1, 2, 3a, 4sa, 4sb, 7, 8a, 9, 11p and 11d [18]. To maintain blood supply to the distal half of the stomach, stations 3b, 4d, 5, and 6 must be preserved. However, if only the antrum is spared, station 4d can be resected, preserving the right gastric artery (station 5), right gastroepiploic artery (station 6), and distal lesser curvature (station 3b) lymph node stations. The omission of the peri-pyloric lymph nodes in PG for LAPGC can be considered oncologically safe, as the risk of metastasis to these nodal stations is consistently low in the literature. A prospective lymphatic mapping study by Kurokawa et al. demonstrates that the risk of metastasis to stations 5 and 6 in GEJ tumors is extremely low, with a cumulative risk of 1%, rising to a cumulative risk of 10% only when tumors extend > 6 cm into the stomach [20]. Similarly, in a study by Yura et al., no metastases were found in stations 5 or 6 among 202 patients with pT2 or T3 proximal gastric cancer [25].

In contrast, the incidence of nodal metastasis to station 4d exceeded the 5% threshold and was reported to be 7.1% in a study by Lee et al., which analyzed data from 878 patients with pT2-4 proximal gastric cancer [26]. Ri et al. further supported this conclusion in their study of 167 cT2-T4 proximal gastric cancers, finding only one metastasis to stations 5 and 6, corresponding to a therapeutic index ≤ 1.4. In contrast, six metastases were observed at station 4d, reinforcing the importance of removing station 4d during PG for LAPGC [27]. The omission of station 3b in LAPGC is also supported by the literature, as regional nodal drainage appears limited distal to the incisura angularis. Haruta et al. reported that while the therapeutic index for station 3a along the proximal lesser curvature was high, the therapeutic index for station 3b distal to the incisura angularis was low, with only a 2.2% metastasis rate in 182 patients [28]. Given the very low reported incidence of peripyloric lymph node metastasis in LAPGC, the oncologic benefit of prophylactic dissection of these lymph nodes with TG is likely negligible as long as certain criteria are met. The previously mentioned Lee et al. multivariable analysis included 878 Korean patients with locally advanced gastric cancer undergoing TG. They included station 4d among the distal lymph node stations (5 and 6) and identified several independent risk factors for metastasis to the distal lymph node stations: proximal gastric tumors with an epicenter more than 3 cm below the GEJ, tumor size over 7 cm, and macroscopic type IV tumor and serosal invasion [26]. Their findings suggest that by including station 4d in the D2 lymphadenectomy and limiting PG to LAGPC with ≤5 cm of gastric involvement, the risk of leaving occult nodal metastases in stations 5 and 6 is negligible.

Dissection of station 10 (splenic hilar lymph nodes) with or without splenectomy can be considered selectively for cancer of the proximal stomach invading the greater curvature (D2+N0) according to JGCTG (6th edition). Lin et al. randomized 536 patients with LAPGC to D2 lymphadenectomy +/− station 10. Although the absolute improvement of 6% in 3-year DFS failed to achieve significance, the authors found that 13.3% of patients had station 10 lymph node metastasis, resulting in a therapeutic index of 8 [29]. In this study, the subgroup of patients with posterior tumors did achieve a survival benefit from station 10 lymphadenectomy. This finding is consistent with other large series and pooled analyses, which report rates of station 10 lymph node metastasis in proximal gastric cancer ranging from approximately 8.8–13.3% in unselected populations, and higher (up to 19–22%) in subgroups with advanced T stage, greater curvature involvement, or posterior wall tumors [30,31,32,33]. The routine inclusion of station 10 lymphadenectomy is somewhat limited by concerns of safety.

However, MIS spleen-preserving splenic hilar (station 10) lymphadenectomy can be safely performed during gastrectomy for proximal gastric cancer by experienced surgeons. Multiple multicenter prospective and randomized trials have demonstrated the feasibility, with morbidity rates comparable to standard D2 dissection and significantly lower than those associated with splenectomy [32,34,35]. Key anatomical considerations for the safe dissection of station 10 include continuing the 11p nodal dissection of the distal splenic artery towards the splenic hilum, ligating and dividing the short gastric arteries at their base (with the vessel sealer). The splenic flexure of the colon should be completely mobilized, and the left gastroepiploic vessels should be ligated at their base as well. The proximity of these nodes to the splenic vessels and pancreatic tail necessitates meticulous dissection to avoid vascular or pancreatic injury.

Moreover, the D2 lymphadenectomy for LAPGC can be further modified to include lower mediastinal lymph node stations. JGCTG (6th edition) recommend including stations 19, 20, and 110 in D2 lymphadenectomy in tumors invading the esophagus. A transhiatal abdominal approach has been recommended for gastric cancers invading less than 3 cm of the distal esophagus based on the results of the JCOG9502 trial [36]. These stations can be safely accessed and removed via transhiatal dissection, as demonstrated by Hirata et al. [21]. A prospective lymph node mapping study reported by Kurokawa et al. found that GEJ adenocarcinomas with ≤2 cm of esophageal involvement had a ≤2.2% risk of metastasis to lower mediastinal lymph nodes in stations 111 and 112, but a higher risk of up to 6% for station 110 [20]. Similarly, Hasegawa et al. reported therapeutic indices of 7.7 and 3.2 for station 110 in Siewert type II and III GEJ tumors, respectively [37]. No other mediastinal lymph node stations exceeded a therapeutic index of 2. Based on this evidence, we propose a modified D2 lymphadenectomy encompassing stations 1, 2, 3b, 4s, 4d, 7, 8, 9, 10, 11p, 11d, and lower mediastinal stations 19, 20, and 110 for LAPGC with ≤2 cm of esophageal involvement and ≤5 cm of gastric involvement as an evidence-based oncologic operation. Our initial experience supports the oncologic adequacy of this approach for LAPGC, as evidenced by negative resection margins in all patients, retrieval of more than 16 regional lymph nodes in accordance with NCCN and AJCC guidelines, and the absence of locoregional recurrences within 18 months. Although our limited follow-up is a limitation of the study, multiple studies consistently report that the majority of locoregional recurrences occur within the first two years following surgery. Specifically, one large retrospective study of patients undergoing curative gastrectomy with D2 lymphadenectomy found a median recurrence-free survival of 16.7 months for local-regional recurrence, with most recurrences occurring within 2 years [38]. Additional data from Western cohorts indicate a median time to recurrence of 12 months, with over 90% of recurrences diagnosed within 2 years [39,40,41]. Since locoregional recurrences are the only pattern of recurrence that is likely to be affected by changing the extent of gastrectomy and lymphadenectomy, we conclude that locoregional recurrence-free survival does provide a reasonable early evaluation of the oncologic safety of the operation.

Additionally, we demonstrated that preservation of only the antrum may offer the same benefit of reducing postoperative weight loss as seen in the CCOG1602 clinical trial, where >50% of the distal stomach was retained [17]. This trial also reported improved quality of life (QoL) in the PG group compared to the TG group. The absence of QoL and patient-reported outcomes in our study is a major limitation and something we hope to remedy in future studies. However, in our experience, the small gastric remnant did not adversely affect bile reflux or gastric emptying. This was not surprising, as studies suggest that the size of the remnant stomach does not impact long-term dietary recovery or nutritional status, as adaptation occurs primarily through increased small bowel motility rather than remnant size [42].

We also demonstrated a trend towards better maintenance of hemoglobin and lower rates of anemia following PG compared to TG. This is consistent with the randomized clinical trial by Park et al. which demonstrated a lower incidence of anemia (20.6%) in the PG with DTR group compared to the TG group (30.4%) and a lower mean reduction in Hgb levels (−5.6% compared to −6.9%, respectively), suggesting a functional advantage in hematologic outcomes after PG with DTR. Although our sample size was limited, the trends seen are promising and are consistent with the literature, warranting further investigation.

Finally, our assessment of reflux esophagitis after PG with DTR was consistent with previous literature. While three patients had mild reflux symptoms that were well-controlled with anti-reflux medications, we demonstrated a low rate (7%) of severe reflux esophagitis necessitating further evaluation with EGD, which is consistent with multiple systematic reviews and meta-analyses that report an incidence of reflux esophagitis between 3.4% and 8.6% at 1 year [43,44]. This suggests that more radical gastrectomy does not negatively affect the incidence of postoperative reflux esophagitis.

## 5. Conclusions

Current literature, along with our preliminary experience, supports the feasibility of standardized PG with modified D2 lymphadenectomy and DTR as an oncologically sound procedure for LAGC. This approach may offer potential benefits, including reduced weight loss and a lower risk of iron deficiency anemia. However, larger studies with extended follow-up are necessary to validate these findings.

## Figures and Tables

**Table 1:** Baseline clinical demographics and pathological factors for patients undergoing proximal gastrectomy (PG), total gastrectomy (TG), and subtotal gastrectomy (SG).

Patient Demographics and Pathological Factors				
	TG (N = 54)	SG (N = 34)	PG (N = 14)	*p*-Value
**Median Age**	63 (IQR 53–69)	66 (IQR 55–76)	64 (IQR 55–69)	0.39
**Sex**				0.40
Female	24 (44%)	17 (50%)	4 (29%)	
Male	30 (55%)	17 (50%)	10 (71%)	
**Median BMI**	26.7 (IQR 24–30)	26.5 (IQR 23–30)	24.6 (IQR 22–27)	0.09
**Clinical Stage (EUS)**				0.42
Stage IB	3 (6%)	6 (18%)	1 (7%)	
Stage IIA	7 (13%)	3 (9%)	0 (0%)	
Stage IIB	13 (24%)	12 (35%)	5 (36%)	
Stage III	19 (35%)	10 (29%)	7 (50%)	
no/nondefinitive staging EUS	12 (22%)	3 (9%)	1 (7%)	
**Diffuse vs. Intestinal**				0.07
Diffuse	20 (37%)	9 (26%)	1 (7%)	
Intestinal	9 (17%)	14 (41%)	3 (21%)	
Missing	25 (46%)	11 (32%)	10 (71%)	
**Signet Ring Cell**	30 (56%)	16 (47%)	1 (7%)	0.01 *
**Siewert Class (GEJ tumors)**				0.26
Type II	6 (12%)	n/a	1 (7%)	
Type III	14 (26%)	n/a	9 (64%)	
**Perioperative Treatment**				0.003 *
Chemotherapy only	32 (59%)	25 (74%)	3 (21%)	
Chemo + Chemoradiation	17 (31%)	5 (15%)	11 (79%)	
Chemo radiation	3 (6%)	3 (9%)	0 (0%)	
Missing	2 (4%)	1 (3%)	0 (0%)	
**Histologic Grade**				<0.01 *
Moderately differentiated	11 (20%)	5 (15%)	4 (29%)	
Poorly differentiated	35 (65%)	25 (73%)	4 (29%)	
unavailable	8 (15%)	4 (12%)	6 (43%)	

* Statistically significant *p* < 0.05.

**Table 2:** Perioperative outcomes for the patients undergoing proximal gastrectomy (PG), total gastrectomy (TG) and subtotal gastrectomy (SG).

Perioperative Outcomes				
	TG	SG	PG	*p*-Value
**Median EBL (mL)**	150	150	175	0.56
**Mean OR time (hours)**	8.5	8.1	7.8	0.78
**Median hospital length of stay (days)**	5	5	4	0.19
**30-day readmission rate (%)**	13.2	23.5	14	0.27
**30-day reoperation rate (%)**	15	8.8	0	0.24
**90-day major morbidity (%)**	26.4	12.9	14	0.28
**90-day mortality (%)**	0	3.2	0	0.34
**R0 achieved (%)**	93	100	100	0.16
**Median LN (IQR)**	30 (25–38)	30 (21–40)	24 (18–38)	0.86
**18-month locoregional recurrence (%)**	7.5	12.5	0	0.50
**18-month overall survival (%)**	77	68	78	0.32

## Data Availability

The data that support the findings of this study are available upon reasonable request from the corresponding author [T.E.G.]. The data are not publicly available due to HIPAA concerns.
